# Highlighting the need for more holistic, participatory design of assistive technologies in displacement settings

**DOI:** 10.3389/fpubh.2026.1675888

**Published:** 2026-03-12

**Authors:** Helen Lindsay, Rashmina J. Sayeeda, Yanina Nahum, Rana Hussein, Carrie Preston, Muhammad H. Zaman

**Affiliations:** 1 Center on Forced Displacement, Boston University, Boston, MA, United States; 2 Department of Biomedical Engineering, Boston University, Boston, MA, United States; 3 Odum School of Ecology, University of Georgia, Athens, GA, United States; 4 Department of English, Boston University, Boston, MA, United States; 5 Women, Gender, and Sexuality Studies Program, Boston University, Boston, MA, United States

**Keywords:** assistive technology, disability, forced displacement, migration, mobility aid, participatory research, refugee health

## Abstract

Forced displacement exacerbates existing disabilities and can lead to the development of functional impairments, especially for women, children, and aging populations. Estimates indicate 15 to 33% of displaced individuals live with disabilities, but data remains limited. This perspective piece describes the current landscape of assistive technologies addressing physical, motor, and sensory disabilities in displacement settings. While some initiatives exist, particularly in mobile technologies and mobility aids, there is a notable lack of targeted development of Assistive Technologies (ATs) and funding opportunities specifically intended for displaced populations with disabilities. Therefore, we emphasize the need for increased attention to this critical topic, and propose a shift toward more robust, ethical, interdisciplinary research initiatives on assistive technologies in displacement settings. Such initiatives should incorporate community-engaged and participatory design, anticipate and mitigate potential harms, and promote dignity and well-being within these communities. We also stress that ATs in displacement settings should be integrated into existing service delivery frameworks rather than remaining as short term or pilot projects.

## 1 Introduction

According to the United Nations High Commissioner for Refugees (UNHCR), 123.2 million people have been forcibly displaced from their homes due to conflict, persecution, and human rights violations (1). Notably, in forced displacement and migration settings, individuals with disabilities are among the most vulnerable and invisible groups, and it is estimated that this group integrates up to 33% of displaced individuals (2, 3). As defined by the International Organization for Migration (IOM), a refugee is a person outside their country, with a “well-founded fear of persecution for reasons of race, religion, nationality, membership of a particular social group or political opinion.” An asylum seeker is a person seeking international protection whose status has not been determined (4). An internally displaced person (IDP) has migrated within their country of origin after being forced to flee their home due to violence or disaster. Stateless persons, those without citizenship in any state, often face similar conditions as other forcibly displaced populations (4). Hereafter, the terms “displaced person” and “displaced populations” will be used to refer to the abovementioned groups, generally excluding individuals who migrate voluntarily for work, educational opportunities, or other personal purposes, although we recognize that people often leave their homes for multiple reasons and the divisions between categories of people on the move are often ambiguous.

The World Health Organization (WHO) defines disability as impairments, limitations and restrictions to activities and participation; a dynamic interaction exists between an individual’s health conditions and both personal and environmental contextual factors. Mobility loss, psychological stress, and inability to access medical aid and education further perpetuate vulnerabilities and disabilities of displaced persons (5). The United Nations Convention on the Rights of Persons with Disabilities ensures the protection of human rights to health equity for all persons with disabilities, which includes access to healthcare and devices that improve quality of life and performance (6). The Social Model of Disability emphasizes that it is not an individual’s physical or mental impairments that disable them (as the Medical Model suggests), but the barriers in the world around them, which limit accessibility and can create prejudice and exclusion (7).

Assistive technologies (ATs) are products and services intended to enhance functional abilities, such as mobility, development and communication, and an overall enhanced experience and improved quality of life for individuals (8). Mindful of the ways societal structures limit accessibility, ATs must be designed to prioritize the dignity and wellbeing of users alongside their functional ability. ATs can include simple technologies, such as glasses or crutches, and more advanced technologies, such as digital tools, software, or 3-D printing devices (9). ATs allow for people with disabilities to access societal, educational, and livelihood opportunities. ATs may be used as preventive measures against acquiring additional impairments and secondary health conditions. Although ATs can be used by a variety of individuals and even the general population, they were originally designed and intended for people with disabilities. Here, we discuss the prevalence of ATs designed for physical, sensory, and motor disabilities among displaced populations. While we recognize the importance of psychosocial disabilities, the focus of this paper is physical, sensory, and motor disabilities which have received limited attention in displacement settings.

Access to assistive technology is a human right, yet the majority of people who would benefit from it lack sufficient access (10). There is a higher prevalence of disability in lower income countries than in higher income countries, due to the disability-poverty cycle, indicating that disability and poverty are both causes and consequences of each other (11, 12). However, ATs are largely designed, accessed, and distributed in high-income countries. Previous reports show that nearly 90% of the people who need ATs have access to them in high-income countries, but only 10% of those in low-and-middle-income countries (LMICs) have access to the AT required (13). Approximately 73% of refugees and additional groups in need of international protection are hosted in low-and-middle-income countries (14). In these contexts, international, local, and regional NGOs provide ATs to fill some of the gaps. United Nations International Children’s Emergency Fund (UNICEF) includes a limited number of ATs—manual wheelchairs and hearing aids—in their supply catalogues (15, 16). The Indian Red Cross Society has provided ATs such as motorized tricycles, wheelchairs, hearing aids, walkers and crutches to people with disabilities. While identified gaps in access persist in LMICs, conflict and displacement settings, access and research evidence on ATs is extremely limited in such complex environments.

Some displaced people with disabilities may have lived with disabilities their whole life, while others have become disabled due to their displacement-causing event, transit, or post-displacement. Physical, motor, or sensory impairments add complications to the ongoing difficulties of being displaced. Healthcare access for displaced populations and in conflict areas is often inconsistent and limited, even unavailable in remote situations (17). This adds to the challenges for displaced populations with disabilities, where existing ATs are commonly unaffordable and out of reach. The sections that follow will provide an overview of current AT initiatives in displacement settings, analyze key trends and gaps, and propose a path forward for future initiatives.

## 2 Current AT initiatives for refugees with disabilities

### 2.1 Academic research on AT accessibility

Access to ATs varies globally, but the lack of ATs designed for displaced people highlights a gap in inclusive humanitarian response. Few studies have assessed the accessibility and use of ATs in refugee-hosting locations. Two studies perform a critical analysis of the existing approaches and policies regarding ATs for disabled and displaced populations, one focused on Italy, and another exploring Istanbul, Türkiye. The article about Italy explored the existing policies for refugees and asylum seekers with disabilities, and strategies to ensure their access to rehabilitation services and ATs. The study concluded that although national policies guarantee healthcare and rehabilitation access to refugees with disabilities in Italy, the system is regionally based and lacks a standardized vulnerability assessment. This can lead to a high degree of variation regionally in the level of support and access for disabled refugees (18). The assessment in Türkiye examining the prevalence of musculoskeletal impairment among Syrian refugees in the Sultanbeyli area found a significant gap between needs and access to health and rehabilitation services and related ATs; the participants of the study reported barriers to AT availability and financial access as the most common barriers (19). In addition to articles exploring access and policies, other literature focused on specific initiatives aimed at improving mobility or access to services. Among these, mobile technology and 3D printing initiatives stood out as the most emphasized approaches in displacement settings.

### 2.2 Mobile technology as ATs in displaced settings

Mobile technologies (MTs) and smartphones have had a tremendous impact on societies in recent decades in accessibility, education, and healthcare, among others (20). In displaced populations, mobile technologies have helped improve communication, integration into host societies, and access to services (21). Limited research has investigated the use of MTs in refugee populations in different regions (22–25), however, existing research has failed to address such technologies designed for refugees with disabilities. One group explored the digital gaps faced by people with disabilities in displaced populations from Kenya, Mali, Ghana, Tunisia and Afghanistan, analyzing the existing interactions between refugees and technology and accessibility and inclusion barriers to MTs (26). The authors called for further research into accessible digital technologies, stressing the need for codesigning with disabled users as an opportunity for the MT sector to engage in initiatives and programs addressing the AT2030 goals, a program working to test solutions to improve access to life-changing ATs for all” (27). One MT initiative intended specifically for disabled refugees in Jordan piloted a telehealth program and assessed its acceptability with community-based rehabilitation workers (CBRWs). During this pilot study, CBRWs were given tablet devices and used a video calling platform to connect with available physical and occupational therapists. CBRWs indicated this process helped clients more quickly achieve therapy-related goals and reduced the long waiting times in the current referral system. The study results demonstrated successful short-term implementation across several domains of feasibility (28).

### 2.3 Mobility ATs in displaced settings

Mobility impairments can compromise the ability to perform daily living tasks, limit employment opportunities, and reduce social interactions (11). For displaced people with disabilities, these challenges are generally potentiated due to inadequate infrastructure, limited healthcare services, and lack of access to ATs. Few academic articles explore the use and design of innovative ATs for displaced populations with disabilities. Three related research projects explored the needs of Rohingya refugees with mobility impairments. In the first study, the researchers used remote monitoring using video footage to identify mobility instability in refugees with disabilities (29). A second study discussed efforts to improve the design of crutches used by refugees with mobility impairments. Surveys of crutch users in Korea and in a refugee camp in Malawi revealed uneven surfaces as a major challenge for users (30). To overcome these challenges, the authors proposed and evaluated a new design for crutch “shoes” that could more easily adapt to uneven surfaces. In the third study, the authors described their use of technology throughout the project, including information and communication tools, computer-aided design, and 3D printing of customized crutch shoes. 3D printing of crutch shoes was found to be an acceptable solution that participants were continuing to use a year after the pilot initiative (31). The authors stressed the importance of on-the-ground presence in “building trust, capturing cultural nuances, and enabling effective co-design with local communities” and noted that technical communication barriers need to be addressed proactively. They also emphasized that sustaining technology access and training post-implementation is key for long-term impact and highlighted the need to incorporate gender dynamics when introducing new technologies.

### 2.4 ATs in non-academic sources

Beyond academic sources, a limited number of innovations designed to improve the lives of displaced and host community members with mobility and sensory disabilities are publicized. Mobility aids include wheelchairs as well as 3D printed prosthetic (artificial limbs) and orthotic (braces) devices. Innovations have been implemented in Bangladesh, Poland (32), Jordan, the Democratic Republic of Congo, South Sudan, Tanzania (33), refugee settlements in Uganda (34), Kenya (35), refugee camps on the border of Thailand and Myanmar, and Lebanon (4) (see Table 1). Among these innovations, two of them were developed to assist people with sensory disabilities, including the development of accessible websites for refugees with visual impairments by Patchwork Observatory and 3D printed hearing aids for refugees with hearing impairments (36). Another project designed wheelchairs that could adapt to sandy terrain, more appropriate for the uneven soils and rough ground of certain refugee camps (37). The AT2030 project, mentioned above, supported 3D printed prosthetic projects in Jordan and other technological initiatives with displaced persons, including provisions of prosthetics in Gaza and to South Sudanese refugees in Uganda (38).

**Table 1:** Examples of ATs designed for displaced populations with disabilities.

Innovation	Designer country	Implementation country	Tech category	Disability category	Direct design engagement with affected communities?
3D Printed Crutch Shoes	Korea	Bangladesh	3D printing	Mobility	No
Patchwork Observatory	Poland	Poland	website	Visual Impairment	Yes—with displaced persons/immigrants, people with disabilities
3D PETRA (Printing through emergency tele rehabilitation access)	UK, France	Uganda	3D printing	Mobility	No—2 local physiotherapists consulted in late stages
3DP4ME (3D Printing for the Middle East)	US	Jordan	3D printing, 3D scanning and modeling	Hearing impairment	No
Comprehensive Community-Based Rehabilitation in Tanzania (CCBRT)	Tanzania	Democratic Republic of Congo, South Sudan, Tanzania	3D Printing	Mobility	No
Mercy corps 3D Printing	US	Jordan	3D printing	Mobility	Yes—consultations with users and medical experts on specific 3D printed projects
Appropriate and Affordable Emergency Wheelchair	UK	Kenya, Philippines, Nepal	Manual wheelchair	Mobility	No
Prosthetics workshops	Thailand/UK	Thailand	Prosthetics	Mobility	Yes—project run by a Burmese Amputee and trained refugees to make prostheses
Dextra	Canada	Morocco, Ghana, Lebanon	3D printing	Mobility	No

Innovations developed for people with disabilities in complex settings may also include humanitarian emergency situations, given the increasing frequency of large-scale natural disasters and rising prevalence of disability in affected populations. For example, ELRHA in partnership with Humanity & Inclusion and Johanniter International collaborated on designing wheelchairs and training packages appropriate for emergency contexts. Supported by the Humanitarian Innovation Fund, the Appropriate and Affordable Emergency Wheelchairs were distributed in Dadaab refugee camp in Kenya, in the Philippines after Typhoon Haiyan in 2013, and in Nepal after the earthquake in 2015 (38).

Adequate funding is critical for the design, implementation, and longevity of ATs that sustainably meet the needs of displaced people with disabilities. The value of the global AT market is projected to reach $31.22 billion by 2030 up from $21.95 billion in 2022 (39). The development of innovations and ATs previously discussed have been sourced by various streams such as UN agencies, collaborations between private companies and international humanitarian aid organizations, non-governmental organizations (NGOs), government funding agencies such as UKAID, and private philanthropic foundations, namely the Grand Challenges Initiatives. Reports from refugees with disabilities in Bangladesh describe that 43% of assistive products are sourced from NGOs and 26% are self-made products (often funded personally or by family/friends) (10). Funding is one area where the support of UNHCR, WHO, National Ministries of Health, and NGOs is key, as it is in the provision of ATs incorporation into service delivery frameworks. While gaps remain in these areas, we focus on design of ATs and encourage the involvement of these organizations as well as researchers, designers, and communities in the process.

## 3 Discussion: shortcomings of existing technological approaches and a proposed path forward

Past efforts to design technologies for displaced and vulnerable populations have made clear that doing so effectively and responsibly requires unique considerations, including a holistic understanding of the relevant sociocultural, historical and political context, refined metrics of success, proactive engagement with questions related to an intervention’s potential to cause harm or exacerbate marginalization and meaningful engagement with affected populations (40). These considerations reveal the shortcomings of relying on traditional engineering paradigms—which tend to measure success in terms of technical functionality, novelty, scalability, and economic outcomes—to guide the development of these technologies (41). While there has been a shift toward prompting engineers to consider their potential to contribute to alleviating complex, global challenges, even well-intentioned projects may contribute to creating or exacerbating harm. Excitement around innovation can sometimes take precedence over ensuring an intervention effectively and responsibly addresses a community’s needs. Techno-solutionism, the belief that even complex human challenges must have simple, technological fixes can mean technological approaches are introduced where they may not be the most appropriate solution, and where instead meaningful interdisciplinary collaboration is needed. In addition, detailed and systematic analyses of current technological solutions, especially their limitations and potential harms in contexts of forced displacement, remain poorly discussed and understood; innovations are often celebrated simply for being novel and technically sound (42) but may fail to address the needs of the community because they did not consider local context nor meaningfully engage affected communities.

Traditional engineering design frameworks, which may overlook local context and fail to proactively consider who an intervention may exclude or marginalize, will naturally lead to designs where disability is an afterthought, if considered at all. Such design paradigms may contribute to explaining the general shortage of ATs developed for contexts of forced displacement. Forced displacement should be understood as a heterogeneous phenomenon encompassing a range of many different contexts and scenarios when developing frameworks (43). A shift toward more ethically grounded and community-centered frameworks based in human rights principles will likely mean disability will naturally emerge as one of many intersecting realities to consider in design processes from the outset. Designing these technologies also requires recognizing the unique considerations needed to effectively support people with disabilities with ATs in displacement contexts, especially in better informing targeted interventions that will support long-term health improvements. Designing ATs for displaced individuals requires shifting the focus away from strictly counteracting or compensating for medical impairments and toward removing social and environmental barriers. This begins with engaging affected communities as co-designers with an active voice rather than as mere recipients of aid, aiming to understand the barriers they face and their root causes. For example, a communication application for individuals with hearing disabilities may be effective in offering sign language interpretations but falls short in addressing broader societal barriers like the lack of trained medical interpreters which may necessitate an app like this in the first place. Critically, as illustrated in the example of crutch “shoes,” it is often insufficient to simply impose existing designs and technologies onto these contexts, where unique challenges must be considered (30). While this case involved the use of 3D printing, future engagement should explore the potential for simpler, low-cost, and resource-light ATs, like the crutch shoes themselves,—designed in conversation with communities who understand the context and challenges best—which can be feasible, scalable and sustainable in humanitarian and displacement settings. In addition, while digital or software tools for remote monitoring of disability may present opportunities for more robust mobility aid analysis, the use of video recordings raises concerns about data privacy, especially in contexts where individuals may be fleeing hostile governments, and particular attention must be paid to preserving their rights and privacy. Designers must consider that communities may be hesitant to accept any unfamiliar technologies and recognize that ultimately, the most effective innovations in these contexts are not necessarily the most novel or technologically complex, but often those that are simple, context-appropriate and which communities themselves can adapt, maintain, and trust.

In addition to the shortcomings identified in aiming to design technologies for displaced communities in general, there are a unique set of considerations that come with designing ATs aiming to support people with disabilities in displacement settings, including:

- How will contextual and environmental factors (e.g., terrain of a refugee camp) affect the feasibility and functionality of the design of traditional assistive technologies?
- Do our efforts to offer specific, catered solutions to a marginalized group present a risk of further marginalizing them?
- Does the need for specific, catered solutions indicate a failure to address broader issues of accessibility and inclusion? Are our designs compensating for the fact that we initially failed to design inclusive services, which proactively considered a broad range of needs from the outset, and therefore must be retroactively adapted?
- Do we assume that incorporating technology in isolation, without considering other barriers to accessibility and systemic challenges, is sufficient?
- What training, resources and support are in place for the community to help ensure meaningful and sustained use of technology? Do we consider that structures of support may look different in displacement contexts?
- To what extent do we consider the role of potential mobility patterns of those who have been displaced in the design of these technologies?
- How do we design and account for sustainability and flexibility in acute crisis settings, historically viewed as short-term situations, but increasingly becoming protracted?

The above considerations point to systemic shortcomings within engineering practice and education, which often do not equip engineers with the ability to holistically approach challenges associated with forced displacement and other complex, global challenges. A shift toward more community-centered, interdisciplinary and ethically grounded engineering approaches would mean many of these questions would naturally emerge to guide the engineering design process. However, while the need for this shift is clear, few formal training opportunities exist for engineering students that would prepare them to responsibly grapple with complex challenges like this one.

## 4 Conclusion

Humanitarian crises and conflicts create an increase in demand for assistive technology, yet the population’s need is far from being adequately met. Currently, 2.5 billion people need at least one form of AT, but in some countries, up to 97% of those in need cannot access them (10). By 2050, more than 3.5 million individuals will require ATs (10). There is currently limited research exploring the needs of individuals with physical, sensory, and motor disabilities in complex, displacement settings and a lack of development of affordable and sustainable ATs, demonstrating a clear need for increased attention to this area. Supporting people with disabilities in these settings must involve the development of research and design frameworks that are ethically guided, informed by the social model of disability, and advised through co-design and participatory approaches with the community.

We call for reimagined research and engineering paradigms, which fundamentally redefine success and failure through a community-centered, human rights lens. This involves proactively anticipating and mitigating potential harms or further marginalization, interrogating not only the need for the technologies we introduce but also whether our efforts risk addressing only surface-level symptoms of deeper inequities, and grounding new technologies in existing social, cultural, and structural dynamics that shape lived realities. Practical steps to do this will, as mentioned, require formal training to be developed for engineering students and extended beyond the school setting, in the form of capacity building for engineers in the field. This training should facilitate the aforementioned paradigm shifts by emphasizing community-centered approaches, ethics and human rights frameworks, critical reflection on existing interventions and interdisciplinary approaches. Through meaningful cross-disciplinary collaboration, the true and ever-evolving needs of displaced communities with disabilities can be effectively and responsibly met. Researchers and NGOs can also move toward using inclusive methodologies and instruments for their data collection to ensure the needs of persons with disabilities are addressed and satisfied during early project phases. Such needs assessments should be participatory, and incorporated into universal and inclusive design approaches. Lastly, ATs should be incorporated into existing service delivery frameworks rather than remaining as short-term *ad hoc* and pilot projects.

The number of products supported by research evidence and sustained by pilot projects is extremely limited. In fact, there is only one example to cite that has been discussed here—the “crutch shoes”—which are also limited to a small population of users (31). We underscore the need for more research on the products needed as well as increased development of accessible ATs, conducted in partnership with displaced communities with disabilities. Translational research, along with ethical context-informed design and involvement of targeted communities in this field can help to close the access gap.

## Data Availability

The original contributions presented in the study are included in the article/supplementary material, further inquiries can be directed to the corresponding authors.

## References

[1] UNHCR. Refugee Data Finder. (2025). Available online at: https://www.unhcr.org/external/component/header

[2] Global Migration Data Analysis Centre. Disability and Human Mobility. (2023). Available online at: https://www.migrationdataportal.org/themes/disability-and-human-mobility

[3] USCRI. Double minority challenge: Immigrants & Refugees with disabilities. USCRI. (2024). Available online at: https://refugees.org/the-double-minority-challenge-faced-by-immigrants-and-refugees-with-disabilities/

[4] International Organization for Migration. Key migration terms. Available online at: https://www.iom.int/key-migration-terms

[5] Akpinar T Arslan ME. Refugees with disabilities: reflections on Turkey and the European Union. The Journal of International Social Research. (2023) 16:1–31. doi: 10.17719/jisr.2023.88174

[6] Who. 10 facts on disability. Geneva. (2023). Available online at: https://www.who.int/news-room/facts-in-pictures/detail/disabilities

[7] Oliver M. Politics of Disablement.SpringerLink. London: Red Globe Press. (1990).

[8] Who. Assistive technology. Available online at: https://www.who.int/news-room/fact-sheets/detail/assistive-technology (Accessed July 28, 2025).

[9] Whittaker G Wood G Oggero G Kett M Lange K. Meeting at needs in humanitarian crises: the current state of provision. Assist Technol. (2021) 33:S3–S16. doi: 10.1080/10400435.2021.1934612 34951828

[10] UNICEF, World Health Organization Global Report on Assistive Technology. Geneva. (2022).

[11] World Health Organization. World Report on Disability. WHO Library Cataloguing-in-Publication Data (2011).

[12] Disability, poverty and development. World Hosp Health Serv. (2002) 38:21–33. 12221831

[13] ATscale Global Partnership. (2025). Available online at: https://atscalepartnership.org/ (Accessed July 28, 2025).

[14] Refugee Data Finder - Key indicators. (2025). Available from: https://www.unhcr.org/refugee-statistics (Accessed September 29, 2025).

[15] UNICEF. Hearing impaired equipment. (n.d.). Available online at: https://supply.unicef.org/all-materials/rehabilitation-disabilities/hearing-impaired-equipment.html

[16] UNICEF. Assistive mobility supply. Available online at: https://supply.unicef.org/all-materials/rehabilitation-disabilities/assistive-technology-mobility.html

[17] Abu El Kheir-Mataria W Tarnas MC Simonek T Al-Jadba G Boatemaa Setrana M Ellis K . Refugee health inclusion: legal, geopolitical, and economic barriers. Int Migr. (2025) 63:e70048. doi: 10.1111/imig.70048

[18] Tofani M Galeoto G Berardi A Iorio S Conte A Fabbrini G . Measuring disability among migrants with Washington group tools: reflections for field use. Healthcare (Basel). (2022) 10:1860. doi: 10.3390/healthcare10101860, 36292309 PMC9601766

[19] Boggs D Atijosan-Ayodele O Yonso H Scherer N O'Fallon T Deniz G . Musculoskeletal impairment among Syrian refugees living in sultanbeyli, Turkey: prevalence, cause, diagnosis and need for related services and assistive products. Confl Heal. (2021) 15:29. doi: 10.1186/s13031-021-00362-9 PMC8056489 33879194

[20] Aranda-Jan CB Casswell J Nique M. "Mobile Technologies as Assistive Technologies in Humanitarian and Development Contexts". In: 2019 IEEE Global Humanitarian Technology Conference (GHTC) (2019). p. 1–5.

[21] Leung L. Telecommunications across borders: refugees’ technology use during displacement. (2010). Available online at: https://opus.lib.uts.edu.au/handle/10453/15479

[22] El-Haj-Mohamad R Nohr L Niemeyer H Böttche M Knaevelsrud C. Smartphone-delivered mental health care interventions for refugees: a systematic review of the literature. Cambridge Prisms: Global Mental Health. (2023) 10:e6. doi: 10.1017/gmh.2022.61, 36843879 PMC9947632

[23] Logie CH Okumu M Berry I Kortenaar JL Hakiza R Musoke DK . Kukaa Salama (staying safe): a pre-post trial of an interactive informational mobile health intervention for increasing COVID-19 prevention practices with urban refugee youth in Uganda. Int Health. (2024) 16:107–16. doi: 10.1093/inthealth/ihad051, 37458073 PMC10759295

[24] Meyer C Surmeli A Hoeflin Hana C Narla N. Perceptions on a mobile health intervention to improve maternal child health for Syrian refugees in Turkey: opportunities and challenges for end-user acceptability. Front Public Health. (2022) 10:1025675. doi: 10.3389/fpubh.2022.1025675 36483243 PMC9722941

[25] Semaan J Farah C Harb RA Bardus M Germani A Elhajj IH. Tackling the COVID-19 infodemic among Syrian refugees in Lebanon: development and evaluation of the “Wikaytek” tool. Digital Health. (2023) 9:20552076231205280. doi: 10.1177/20552076231205280, 37915792 PMC10617281

[26] Oliver M. Understanding Disability: From Theory to Practice. New York: Bloomsbury Publishing (2018). p. 208.

[27] AT. AT2030 Programme. (2020). Available online at: https://at2030.org/at/

[28] Mitchell-Gillespie B Hashim H Griffin M AlHeresh R. Sustainable support solutions for community-based rehabilitation workers in refugee camps: piloting telehealth acceptability and implementation. Glob Health. (2020) 16:82. doi: 10.1186/s12992-020-00614-y, 32933537 PMC7491020

[29] Brown S Hussain F Vairis A Hacker E Bess M. Remote monitoring of disability: a case study of mobility aid in Rohingya camp. (2022). Available online at: https://link.springer.com/chapter/10.1007/978-3-030-98404-5_10 (Accessed July 28, 2025).

[30] Brown S Vairis A Masoumifar A Petousis M. Common problems with the conventional design of crutches: proposing a safer design and discussing the potential impact. Technol Soc. (2020) 60:101215. doi: 10.1016/j.techsoc.2019.101215

[31] Hussain F Brown S. Ict tools for addressing mobility needs of rohingya refugees with disabilities: practical challenges and solutions. Inf Technol Dev. (2024) 30:644–64. doi: 10.1080/02681102.2024.2327864

[32] Service UI. Refugees with disabilities advancing inclusion and self-reliance. UNHCR innovation service. (2024). Available online at: https://medium.com/unhcr-innovation-service/refugees-with-disabilities-advancing-inclusion-and-self-reliance-be9349c04b48

[33] Grand Challenges Canada. Comprehensive Community-Based Rehabilitation in Tanzania (CCBRT). (2019). Available online at: https://humanitariangrandchallenge.org/innovator/mobility-for-all/ (Accessed July 28, 2025).

[34] Barker S. 3D printing, a game changer for humanitarian innovation | Humanity & Inclusion Canada. (n.d.). Available online at: https://www.hi-canada.org/en/news/-3d-printing--a-game-changer-for-humanitarian-innovation- (Accessed July 28, 2025).

[35] ELRHA. Latest small grant award: appropriate and affordable emergency wheelchairs. (2011). Available online at: https://www.elrha.org/news-blogs/latest-small-grant-award-appropriate-affordable-emergency-wheelchairs (Accessed July 28, 2025).

[36] 3DP4ME. Empowering patients to walk, move, and hear with 3D printed prostheses and hearing aids. (2017). Available online at: https://3dp4me.org/

[37] Home | AT2030 Programme. Available online at: https://at2030.org/#gsc.tab=0 (Accessed July 28, 2025).

[38] World Economic Forum. How sovereign funds could empower the future of assistive technology and disability AI. (2023). Available online at: https://www.weforum.org/stories/2023/08/sovereign-funds-future-assistive-technology-disability-ai/ (Accessed July 28, 2025).

[39] Wood G Whittaker G. Assistive technology in humanitarian settings | Innocenti Global Office of Research and Foresight. (2022). Available online at: https://www.unicef.org/innocenti/reports/assistive-technology-humanitarian-settings (Accessed September 29, 2025).

[40] Arshad-Ayaz A Naseem M Mohamad D. Engineering and humanitarian intervention: learning from failure. J Int Humanit Action. (2020) 5:7. doi: 10.1186/s41018-020-00073-5

[41] Wiley.com. Engineering Justice: Transforming Engineering Education and Practice. Hoboken: Wiley.

[42] Sandvik KB Gabrielsen Jumbert M Karlsrud J Kaufmann M. Humanitarian technology: a critical research agenda. Int Rev Red Cross. (2014) 96:219–42. doi: 10.1017/S1816383114000344

[43] Ching C Sayeeda RJ Tarnas MC Gross N Abu-Khalil A Shaikh B . Mapping utility and applicability of research and ethics frameworks for displaced populations. Glob Public Health. (2025) 20:2557322. doi: 10.1080/17441692.2025.2557322, 40975798

